# Chitosan-Based Composite Materials for Prospective Hemostatic Applications

**DOI:** 10.3390/md16080273

**Published:** 2018-08-04

**Authors:** Zhang Hu, Dong-Ying Zhang, Si-Tong Lu, Pu-Wang Li, Si-Dong Li

**Affiliations:** 1 Department of Applied Chemistry, School of Chemistry and Environmental Science, Guangdong Ocean University, Zhanjiang 524088, Guangdong, China; 17875128648@163.com (D.-Y.Z.); smelilst@163.com (S.-T.L.); 2 Agricultural Product Processing Research Institute, Chinese Academy of Tropical Agricultural Sciences, Zhanjiang 524001, Guangdong, China; puwangli@163.com

**Keywords:** chitosan, composite materials, hemostasis, mechanisms, films, sponges, hydrogels, particles, fibers, applications

## Abstract

Effective hemostasis is vital to reduce the pain and mortality of patients, and the research and development of hemostatic materials are prerequisite for effective hemostasis. Chitosan (CS), with good biodegradability, biocompatibility and non-toxicity, has been widely applied in bio-medicine, the chemical industry, the food industry and cosmetics. The excellent hemostatic properties of CS have been extensively studied. As a result, chitosan-based composite hemostatic materials have been emerging. In this review, the hemostatic mechanism of chitosan is briefly discussed, and then the progress of research on chitosan-based composite hemostatic materials with multiple forms such as films, sponges, hydrogels, particles and fibers are introduced. Finally, future perspectives of chitosan-based composite hemostatic materials are given. The objective of this review is to provide a reference for further research and development of effective hemostatic materials.

## 1. Introduction

Hemostasis is a vital step in emergency medical care. Effective and quick hemostasis is critically important for surgical operation and emergent trauma, particularly for trauma caused in battlefields and other complicated situations [1,2]. Hemostatic materials currently available from the market mainly include collagen (Col), gelatin (GE), alginate (AG), chitosan (CS), oxidized cellulose, cyanoacrylic acid tissue adhesive and porous zeolite. All of these have effective hemostasis functions, but there are some disadvantages as well. For examples, collagen has limited hemostatic efficacy because it relies solely on activating platelets to stop bleeding and has poor tissue adhesion [3]. Porous zeolite will release a great amount of heat when it absorbs moisture from the blood, leading to wound inflammation [2]. Carboxymethyl cellulose dressing cannot be degraded in the wound and easily causes scars when being removed. Recently different hemostatic agents have been developed, but most of them are ineffective in stopping severe bleeding and expensive or cause safety concerns [4]. Thus, there is great interest in the development of novel effective hemostats to achieve hemostasis.

CS is a natural polycationic polysaccharide which can be obtained from different sources, e.g., shrimp, crab, squid and certain fungi. It is a multi-functional material with good biocompatibility, no immunogenicity and no skin irritation. In 2001, it was approved by Food and Drug Administration of the United States (FDA) as a GRAS (Generally Recognized as Safe) substance [5,6,7]. Currently, a number of FDA-approved chitosan-based hemostatic products including Celox^®^ (MedTrade Products Ltd., Cheshire, UK) TraumaStat^®^ (Ore-Medix, LLC Company, Lebanon, OR, USA) and HemCon^®^ Bandage (HemCon Medical Technologies Inc., Portland, OR, USA) are commercially available. However, it still is a challenge to enhance their hemostatic potential. CS-based composite hemostatic materials refer to a series of novel multi-effect hemostats prepared by combining physically and chemically modified CS and its derivatives with other functional materials. Composite materials have attracted much attention due to their potential synergistic effects that can result in high performance. Therefore, CS-based composite hemostatic materials are becoming more and more extensive in applications. So far, many novel CS-based composite hemostatic materials have been proved to be effective in fast hemostasis and functional hemostasis. This paper reviews the existing studies on CS-based composite hemostatic materials and attempts to provide a reference for further research and development of the novel hemostatic materials.

## 2. Hemostatic Mechanisms of Chitosan (CS)

As early as 1964, the waterfall theory of blood coagulation was put forward, which laid the foundation for the study of endogenous coagulation pathways. The conventional hemostats facilitate blood clotting by activating a certain aspect described in the waterfall theory. However, many studies showed that chitosan triggered coagulation without the activation of the intrinsic pathway, indicating that the hemostatic mechanism of CS was independent of the classical coagulation cascade [7,8,9]. Although it was not quite clearly understood yet, the hemostatic mechanism of CS involved the following aspects.

### 2.1. Aggregation of Red Blood Cells

The red blood cell (RBC), the main kind of hematocyte in the blood, increases blood viscosity and enhances the transportation of platelets to the vascular wall for the physiological hemostasis. On the surface of the RBC cell membrane, there are various proteins and glycolipids that are negatively charged. In a physiological state, the aggregation and adhesion of RBC are inhibited owing to electrostatic repulsion [10]. CS is a natural cationic alkaline polysaccharide; the positive charge of -NH_3_^+^ on CS chain electrostatically interacts with the anions on the surface of RBC, leading to intensive aggregation of RBC around the wound site to form blood clots which quickly stop bleeding [11]. Therefore, the degree of protonation of amino groups on chitosan chains plays an important role in the adsorption of red blood cells. Studies have shown that the ability of chitosan to initiate coagulation was related to the percent of deacetylation, and was more dependent on the number of protonated amine groups [12,13]. Additionally, the interaction of chitosan with red blood cells increased with the increase of its molecular weight, which might be explained by the increase of entanglement degree due to the special intermolecular hydrogen bonding force or electrostatic repulsion between polyelectrolyte molecules [14,15].

### 2.2. Stimulation of Platelets

Under normal physiological conditions, platelets do not adhere to endothelial cells. However, in wounds, the activation of platelet adhesion and aggregation plays a vital role in the process of hemostasis [16]. Biopolymers can trigger the activation of platelet adhesion and aggregation, which is a complex process that is dependent on a variety of properties including surface chain mobility, surface chemical composition, hydrogen bonding properties, charge density, hydrophobicity/hydrophilicity, and so on [17]. Some studies showed that CS could enhance the activation of platelets and accelerate the adhesion and aggregation of platelets [18]. Sagnella et al. [19] found that neutralization of the positive charge on chitosan resulted in a decrease in the number of adherent platelets and aggregates and no significant effect on coagulation activation indicating that a high positive charge density in chitosan was necessary to cause an increase in platelet adhesion and aggregation.

### 2.3. Contact System Activation

**Figure 1:**
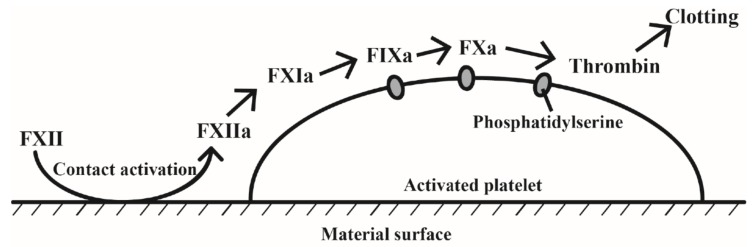
Simplified scheme of blood clotting reactions on material surface (FXIIa, FXIa, FIXa and FXa are for activated coagulation factors XII, XI, IX and X, respectively).

The contact of biomaterial surface with blood directly affects blood coagulation by modifying the protein function after adsorption, which is called the contact activation pathway [20]. This pathway includes the coagulation factors XII (FXII), coagulation factors XI (FXI), high-molecular-weight-kininogen (HMWK), and pre-kallikrein. Contact of blood with functional biomaterials may produce two biological processes, including platelet adhesion and contact system activation, which have a strong synergistic effect on biomaterial-induced blood coagulation (Figure 1) [21]. Studies showed that CS induced blood coagulation in vitro was independent of coagulation factors VII (FVII) but required FXI and FXII [22]. In addition, Fischer et al. [23] found that CS had a series of biochemical reactions in vivo by combining nearly all plasma proteins and some of the important blood coagulation factors, and thus further strengthened the blood clots. However, studies concerning the inactivation of the contact system on the chitosan surface were also reported [8], and the conclusions are still controversial.

### 2.4. Formation of Spatial Network Structure

**Figure 2:**
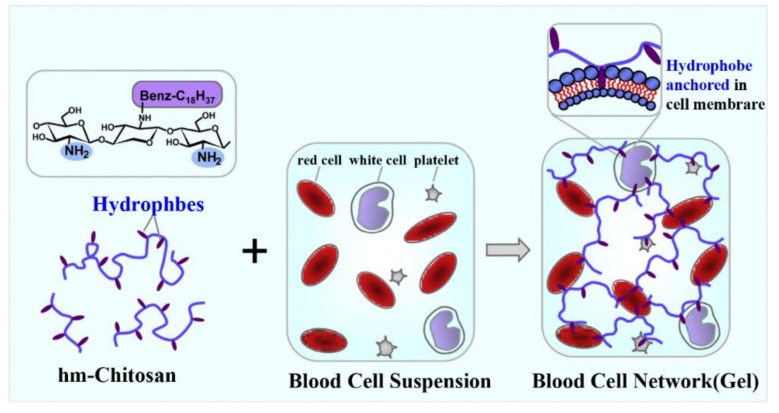
Mechanism for gelation of blood by hydrophobically modified (HM) chitosan (CH).

Considered from the point of view of molecular structure, CS is a linear glycosaminoglycan which makes it easy to construct a network structure, thus promoting the interaction of blood components with CS and facilitating formation of strong blood clotting. A gelling hemostatic mechanism was proposed by Dowling et al. on the self-assembly of hydrophobically modified chitosan (HM-CS) with the blood cells [24]. Once hydrophobes of HM-CS contacted the blood cells, they anchored into the hydrophobic interiors of blood cell membranes via hydrophobic interactions. As a result, a three-dimensional gel network was bridged between the CS chains and blood cells, which could potentially halt the flow of blood (Figure 2).

## 3. CS-Based Composite Hemostatic Materials

Owing to its superior properties such as hemostasis, antibacterial, anti-inflammatory, wound healing, biocompatibility, biodegradability and non-toxicity, CS was expected to be an excellent hemostatic agent [25,26,27]. Nevertheless, hemostatic materials with CS alone have limited hemostatic effects. To improve the hemostatic performance, CS-based composite materials have been prepared by blending CS with other functional components, which can act synergistically to realize fast and effective hemostasis. As chitosan can be easily processed into various forms, CS-based composite materials have been made into a variety of forms, such as films, sponges, hydrogels, particles and fibers. In recent years, more and more chitosan-based functional materials have been developed. In the present review, chitosan-based composite materials for hemostasis are summarized (Table 1).

### 3.1. CS-Based Composite Hemostatic Films

**Figure 3:**
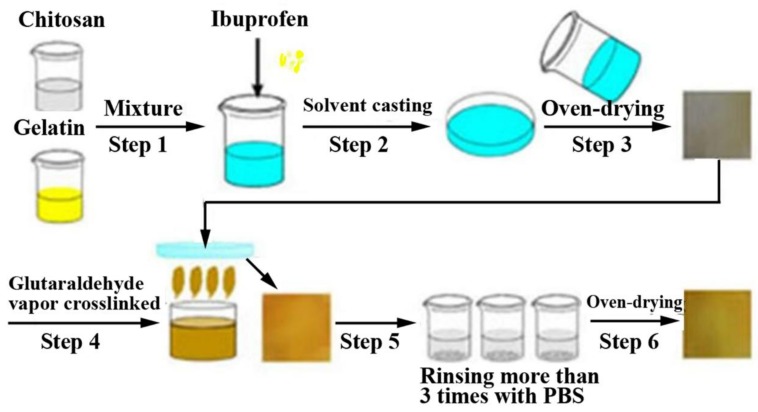
Preparation scheme for cross-linking of drug-loaded CS/GE composite films.

In recent years, CS and gelatin (GE) have been generally used as the primary components of hemostatic dressings [57,58,59,60,61,62]. CS is an abundant natural alkaline polysaccharide that can accelerate blood coagulation and facilitate tissue growth and wound healing. GE is a macromolecular hydrophilic colloid obtained by partial hydrolysis of collagens, and has been widely used in food, cosmetics, medicine and other fields due to its unique physical-chemical properties. Li et al. [28] prepared ibuprofen-loaded chitosan/gelatin (CS/GE) composite films (Figure 3) using the solvent casting method. The results indicated that the amount of CS in the composite films directly affected the tensile strength and elongation at break. The cross-linking of glutaraldehyde was likely to increase the moisture vapor transmission rate and the swelling degree of the composite films. As shown in the assays of antibacterial activity against *Escherichia coli* and *Staphylococcus aureus*, the ibuprofen-loaded CS/GE composite films had better antibacterial effects against the latter. Moreover, the cell counting Kit-8 (water-soluble tetrazolium-8) assay confirmed that CS/GE composite films had no obvious cytotoxicity on living cells, and the assessment of hemostatic effects indicated that the ibuprofen-loaded CS/GE composite films could reduce bleeding in a surgical operation with low pressure and had good absorbance.

In order to achieve a satisfactory hemostasis effect, the selection of suitable components for preparing powdery hemostat is essential. Alginate (AG) is a kind of natural linear polysaccharide extracted from brown algae. Because of its biocompatibility, low toxicity and low price, AG has been widely applied in the fields of biological carriers and tissue engineering. In the application of hemostatic materials, AG has attracted increasing attention due to its superior performance such as excellent adhesion to wounds and high water absorption capacity [63,64]. *Yunnan Baiyao*, a well-known herb prescription used in oriental countries for more than 100 years, is an effective surgical sealant and hemostat. However, it is not suitable for direct intraluminal application due to the lack of an ideal substrate [21,65,66]. Lu et al. [29] prepared chitosan/sodium alginate-*Yunnan Baiyao* composite hemostatic films by blending *Yunnan Baiyao* with CS and sodium alginate. A liver bleeding model of rats was established and the hemostatic effects of the films were analyzed by semi-quantitative evaluation. Compared with the chitosan/sodium alginate composite film group, CS hemostatic film group and GE sponge group, chitosan/sodium alginate-*Yunnan Baiyao* composite films had better hemostatic effects, indicating that *Yunnan Baiyao* acted synergistically with CS and sodium alginate to exert stronger hemostasis.

Mesoporous bioactive glass (MBG) is a promising new family of biomaterials. Owing to its uniform nanoscale mesoporous structure, high specific surface areas and good biological activities, MBG can be used as a good hemostat [67,68,69,70]. However, there are still some problems to be solved in the hemostatic process of MBG. Jia et al. [30] prepared MBG/CS composite porous films successfully by the freeze-drying technique. The MBG/CS films were highly porous with continuous structure of well-interconnected pores and had good water adsorption which could be modulated by varying the mass ratio of MBG and CS. In the liver hemorrhage model of rats, the composite films with different MBG/CS ratios had varied hemostatic effects. When increasing MBG, the hemostatic time and amount of bleeding decreased. In vitro degradation behavior indicated that the composite films had good degradability. Meanwhile, the composite films showed good biocompatibility and non-cytotoxicity. Compared with many other hemostatic materials, MBG/CS composite porous films had the potential to become a novel hemostatic material.

Pourshahrestani et al. [71] found that the capability of platelet aggregation, thrombus formation, and blood coagulation activation of MBG and its biodegradability and biocompatibility can be enhanced by tuning the composition of a MBG with incorporation of low concentration of therapeutic gallium ions (Ga^3+^) into its matrix. On the basis, Ga-MBG/CS composite scaffolds containing various concentrations of Ga-MBG were constructed using the lyophilization method and their properties and hemostatic efficacy were assessed [31]. The results demonstrated that Ga-MBG/CS scaffolds with high porosity exhibited increased water uptake compared to that of commercially available Celox rapid gauze (CXR). CS and Ga-MBG had a synergistic effect, which endowed the GA-MBG/GS composite scaffolds with higher hemostatic efficacy, more significant antibacterial activity, and better biocompatibility than CXR. Therefore, the novel Ga-MBG/CS composite materials could be promising hemostatic candidates for clinic applications.

### 3.2. CS-Based Composite Hemostatic Sponges

As promising biomaterials for hemostasis, biodegradable sponges have been clinically required in the past decades [72]. GE has been extensively studied as an absorbable hemostatic material for its superior properties such as low antigenicity, good biocompatibility and biodegradability [73,74,75]. GE is water soluble and can be blended with many natural polymers to improve the chemical stability and mechanical performance of composite materials. Many composite functional materials were fabricated by crosslinking GE with CS [76,77,78]. Lan et al. [32] prepared a CS/GE composite porous sponge by a modified gradual-base extraction and freeze drying using tannic acid as a crosslinking agent. The in vitro blood coagulation experiments showed that the hemostatic efficacy was optimum at a CS/GE ratio of 5/5 (*w/w*). In the rabbit ear artery hemorrhage and liver laceration model hemostasis experiments, it was found that the hemostatic efficacy of CS/GE sponge was obviously better than that of the material with CS or GE alone, and in regard of liquid absorption and platelets agglutination, the composite sponge was better than the two components separately. Cell experiments indicated that CS/GE sponge could induce cellular proliferation and no significant cytotoxicity. Antibacterial experiments indicated that CS/GE composite sponge had obvious bacteriostatic effects on *Escherichia coli* and *Staphylococcus aureus*. CS/GE composite sponge was expected to be applied as a biological material for surgical hemostasis.

Sepia ink has been used for centuries in Chinese traditional medicine due to its many bioactivities, such as antiradiation, antitumor, immunomodulatory activity and procoagulant function [79]. In our preliminary experiments, squid ink polysaccharide (SIP) could shorten the blood coagulation time in vitro and in vivo, and also activate coagulation factor FXII, indicating a significant procoagulant effect. To overcome the limited hemostatic effects of chitosan, SIP-CS composite sponges were developed by employing the freeze drying technique [33]. SIP-CS sponges optimized by response surface methodology could adsorb hemocytes and stop bleeding rapidly. Compared with chitosan, the addition of SIP improved the hemostatic efficacy of composite sponges. In scalded New Zealand rabbits, SIP-CS sponges could also promote wound healing, reepithelization, and repair of the epidermis and dermis. Therefore, SIP-CS composite sponges were novel marine biomaterials for rapid hemostasis and wound healing.

As a new biomedical material, it is necessary to detect its biological safety. To examine whether hemostatic sponge materials have negative effects on blood systems, Zhang et al. [34] prepared chitin and sepia ink hybrid hemostatic sponge (CTSH sponge) by a freeze-drying process and implanted it into the abdominal subcutaneous layer of mice. The results showed that CTSH sponge had no significant effects on the blood parameters of mice including coagulation, anticoagulation, fibrinolytic and hemorheology, suggesting that CTSH sponge had potential possibility to be developed into clinical hemostatic agents.

Hydroxybutyl chitosan (HBC) is a derivative of chitosan derived by conjugating hydroxybutyl groups to the chains of chitosan. It has good water solubility and controlled temperatue-sensitive properties which made the phase transformation process of the hydrogel reversible [80]. To make up for the drawbacks of pure chitosan sponge, Hu et al. prepared a composite sponge by physically mixing HBC with CS through vacuum freeze-drying [35]. The HBC/CS sponge possessed high porosity (about 85%), great water absorption (about 25 times), good softness, no cytotoxicity and excellent antibacterial properties. In vitro blood clotting studies showed that the HBC/CS composite sponge could make the blood form viscous gels which were conducive to promoting blood coagulation. Rat wound-healing experiments showed that the composite sponge could support the creeping growth of epithelial cells to promote wound healing. 

Proper blood coagulation at the wound site is a prerequisite for wound healing, and the excellent properties of a surgical hemostat play a vital role in the process. Oxidized nanofibrillar cellulose (ONFC), a derivative of cellulose which is the most abundant natural linear polysaccharide, is biocompatible and bioabsorbable [81]. It absorbs water from the blood to form hydrogels causing plasmatorrhexis of the adjacent red blood cells and the activation of platelets [82]. In order to obtain a more effective hemostatic agent, Sukul et al. [36] fabricated a novel ONFC-CS composite hemostatic sponge by linking the carboxyl group (-COOH) of ONFC with the amino group (-NH_2_) of CS via peptide bonds to form a stable hydrogel network that required no addition of cross-linking agents. Cytotoxicity and cell proliferation studies revealed that ONFC-CS hemostatic sponge was non-cytotoxic and the cells proliferated continuously on the sponge. Hemostatic evaluation of ONFC-CS sponge on hepatic trauma indicated that ONFC-CS sponge had better hemostatic effects than ONFC sponge. The combination of ONFC and CS produced the synergistic hemostatic actions. The in situ crosslinked hydrogel network also provided a stable framework for fast absorption of blood and facilitated blood clotting. A rat hepatic trauma implantation experiment showed that ONFC-CS sponge possessed superior biocompatibility and biodegradability. These findings indicated that ONFC-CS sponge was a potential surgical hemostat.

**Figure 4:**
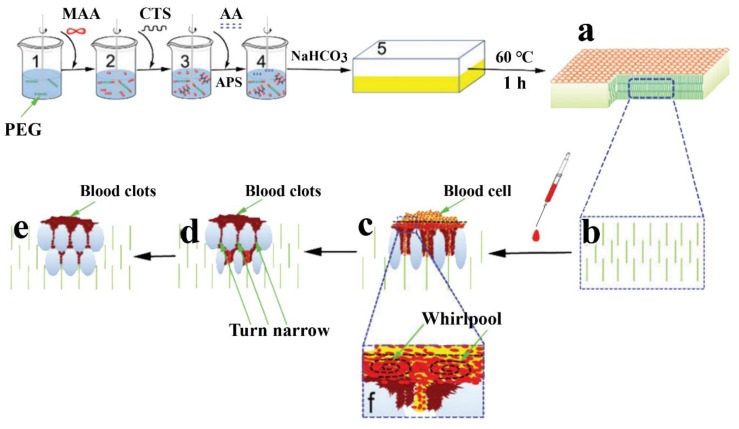
Synthesis and schematic diagram of hemostasis mechanism of hemostatic sponge: (**a**) SPA-co-CTS xerogel sponge was abtained; (**b**) The sponge was with tapered channels ranging within 200–500 mm in diameter; (**c**) The blood that flowed into the tapered channels of the sponge was soon concentrated; (**d**) The wall of the channels became progressively thicker and the channels were narrowed; (**e**) The channels were further narrowed until closed and the blood was concentrated to induce clotting; (**f**) The water in the blood was quickly absorbed so as to produce the blood whirlpools.

Rapid deprivation of water from blood causes blood cells and coagulation factors to concentrate, triggering blood clots. A new hemostatic system that contained covalently bonded chitosan, sodium polyacrylate (SPA) and polyethylene glycol (PEG) in a porous network was developed by Qian et al. [37]. By a simple one-pot reaction (Figure 4), they fabricated a soft, elastic porous SPA-co-CS xerogel sponge which could reach maximum water absorbency of 180 in less than 200 s. In thromboelastography (TEG^®^) test and in a rabbit lethal extremity arterial bleeding model, SPA-co-CS sponge demonstrated superior hemostatic effects by concentrating platelets and exerting its hemostatic effects in a dynamic thrombosis manner. Compared with the existing commercial products such as QuikClot zeolite granules (Z-Medica Corporation, Wallingford, CT, USA), QuikClot Combat Gauze (Z-Medica Corporation) and Celox (MedTrade Products Ltd., Cheshire, UK), SPA-co-CS sponge also displayed significantly enhanced properties of wound sealing, external pressure application, and removal after use.

**Figure 5:**
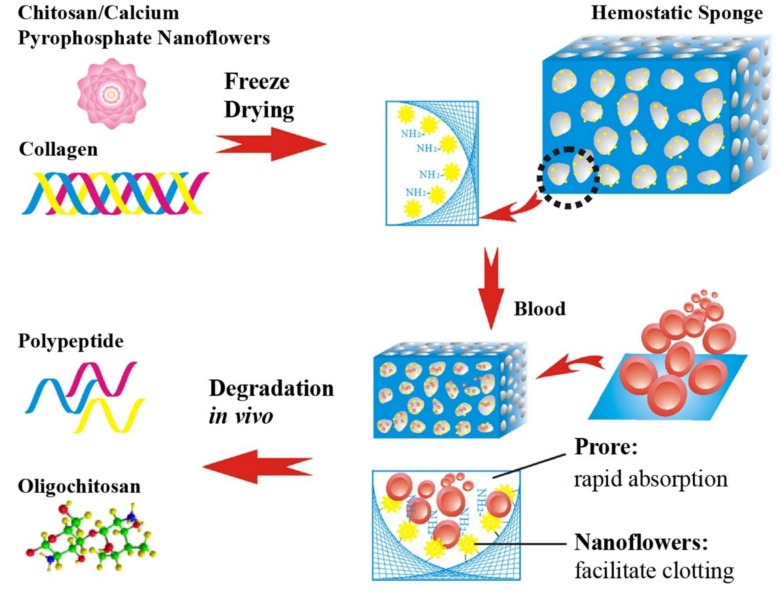
Schematic illustration of the preparation process of hemostatic sponge and mechanism for its degradation.

Organic–inorganic hybrid nanoflowers have been applied in many fields, including drug delivery [83], enzyme immobilization [84] and chemical testing probes [85,86]. Since it is difficult to achieve desired effects from the traditional hemostatic materials, especially in parenchymal organs with rich vascularity, to realize rapid hemostasis Yan et al. [38] synthesized a biodegradable collagen sponge reinforced with chitosan/calcium pyrophosphate nanoflowers (CPNFs-Col) which was prepared by an improved one-pot-preparation method (Figure 5) [87]. The CPNFs-Col hemostatic sponge, with advantages such as rapid water absorption ability, a surface rich in amino groups, and high specific surface areas, could activate the intrinsic pathway of a coagulation cascade, induce haemocytes and platelets adherence, promote blood clotting and achieve rapid hemorrhage control. Additionally, CPNFs-Col sponge could be completely biodegraded in three weeks and thus was suited for post-operative treatment and the prevention of peritoneal adhesion.

The traditional preparation methods of sponges mainly focus on particle leaching, electrospinning, gas foaming and freeze-drying. However, there exist some shortcomings in these methods, such as organic solvent residue and special temperature requirements. In the past few decades, supercritical fluid (SCF) has emerged as an effective alternative to the traditional manufacturing processes and has been widely applied in biomedical applications [88]. Composite sponges with the chemical compositions of CS and poly-(methyl vinyl ether-co-maleic anhydride) (PVM/MA) were successfully prepared by using ammonium bicarbonate particles as a porogen in a supercritical CO_2_ process [39]. The CS-PVM/MA sponges possessed a porous structure (about 80% porosity) that allowed red blood cells to form erythrocyte clots or plugs. In vitro and in vivo experiments indicated that the CS-PVM/MA sponges had a strong clotting ability similar to that of Avitene, a commercially available collagen hemostat.

### 3.3. CS-Based Composite Hemostatic Hydrogels

Hydrogels derived from polysaccharides have unique advantages, such as excellent biodegradability and biocompatibility, high swelling capacity, rapid hemostasis and providing a moist environment. Therefore, they have attracted more and more interest [89].

**Figure 6:**
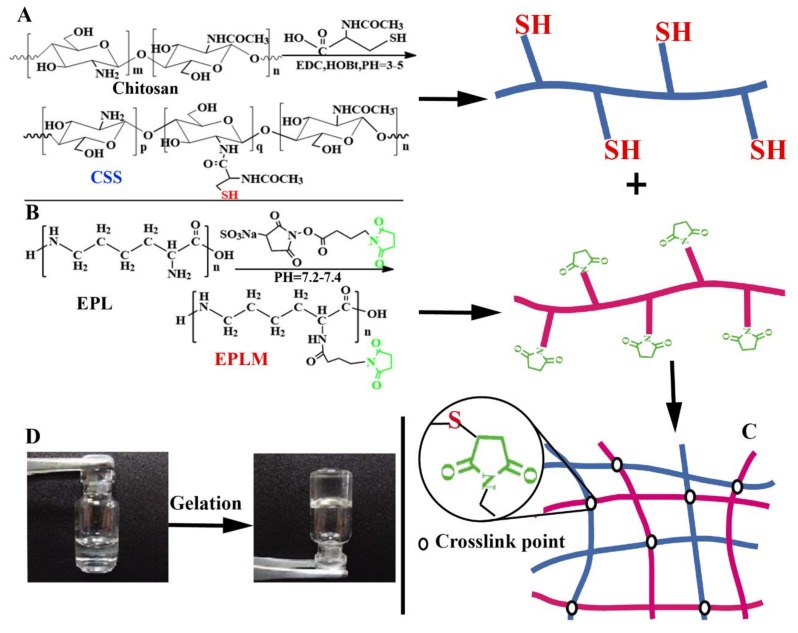
Schematic representation of the synthesis of crosslinking thiol functionalized chitosan (CSS) (**A**); and the synthesis of maleimide group modified ε-polylysine (EPLM) (**B**); demonstration of the in situ hydrogel formation (**C**); photographs of hydrogel formation (**D**).

The Michael addition reaction is one of the most attractive and powerful methods in the modern organic synthesis of valuable compounds. Since it can happen by simply blending the starting materials under mild conditions without any by-products, Michael addition is a better choice for in situ formation of biocompatible hydrogels [90,91]. Nie et al. [40] developed a novel polysaccharides/polypeptide hydrogel for application as adhesive sealant and hemostatic material by fast in situ crosslinking thiol functionalized chitosan (CSS) with maleimide group modified ε-polylysine (EPLM) via Michael addition under mild conditions (Figure 6). Due to similarity with the natural extracellular matrix, the CSS/EPLM hydrogel showed no cytotoxicity. In vivo hemostatic ability test demonstrated that the CSS/EPLM hydrogel possessed excellent hemostatic property.

**Figure 7:**
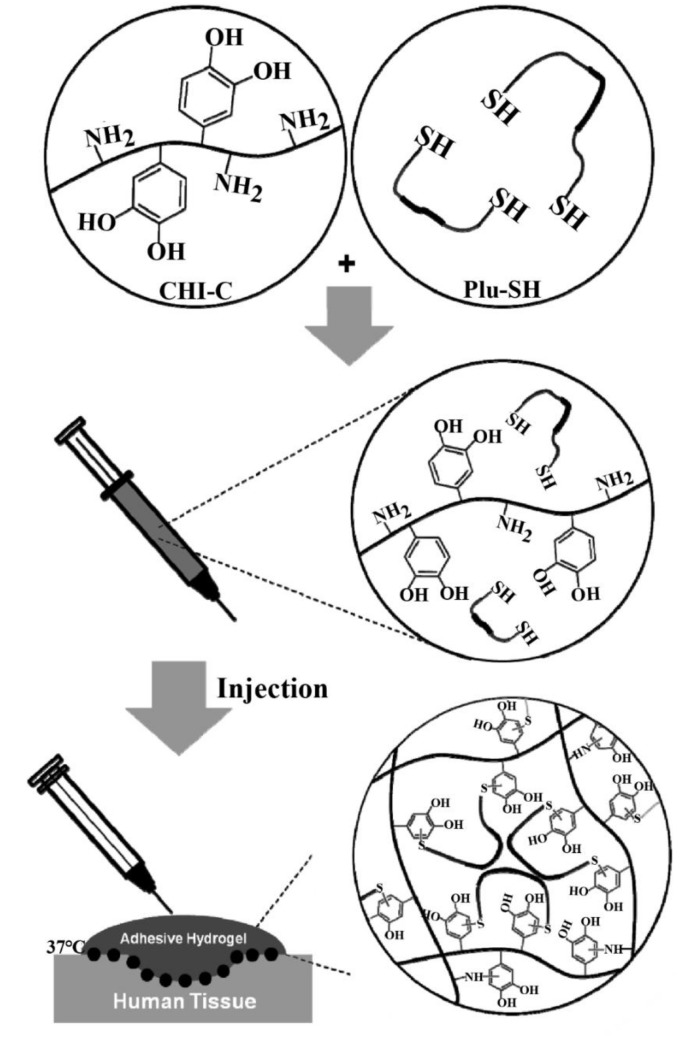
Schematic representation of tissue adhesive, thermosensitive, and in situ cross-linkable chitosan/Pluronic (CS-C/Plu-SH) hydrogels.

Tissue adhesive materials have been widely used in the fields of wound healing, surgical tissue adhesives, wrinkle fillers and hemostasis in surgical operations [92,93]. From the adhesion behaviors of mussels, it was found that mussel adhesive protein played a critical role in forming hydrogel-like adhesive pads on the substrates. Bioinspired from the mussel adhesion mechanism, Ryu et al. [41] synthesized injectable and thermosensitive chitosan/Pluronic hydrogels (CS-C/Plu-SH) for tissue adhesives and hemostatic materials by in situ crosslinking between catechol-functionalized chitosan and thiol-terminated Pluronic (Figure 7). In vivo and in vitro evaluation indicated that CS-C/Plu-SH hydrogels had superior mechanical performance and stability. Tissue adhesion assessment and rat hemorrhaging liver model indicated that CS-C/Plu-SH hydrogels had strong adhesiveness to soft tissues and mucous layers and also showed excellent hemostatic properties.

Human-like collagen (HLC) is a novel genetically engineered protein expressed by recombinant *Escherichia coli* BL21 (O7. expression molecular biology strain with T7 polymerase for pET system) containing a partial cDNA clone obtained from reverse transcription from human mRNA. In addition to the characteristics of collagen, HLC has many superior properties, such as excellent water solubility, low immunogenicity, good stability and non-toxicity. Therefore, HLC has been considered a promising biomaterial [94]. Pan et al. developed a series of soft, flexible, porous, translucent, breathable, non-stick hydrogel dressings through a simple repeated freeze-thawing process [42]. By simply mixing the solutions of poly (vinyl alcohol) (PVA), HLC and carboxymethyl chitosan (CMCS) and adding Tween 80 as a pore-forming agent, PVA-HLC-CS-T80 hydrogels were successfully fabricated. The overall results showed that PVA-HLC-CS-T80 hydrogels presented excellent swelling ratios, bacterial barrier activity, moisture vapor permeability, hemostasis activity and biocompatibility. Additionally, in vivo evaluation revealed that PVA-HLC-CS-T80 hydrogels significantly enhanced wound healing by reducing inflammation, promoting granulation tissue formation, collagen deposition and accelerating re-epithelialization. These results indicated that PVA-HLC-CS-T80 hydrogels had great potential as dressings for hemostasis and wound healing. By using radiation technology, Fan et al. [43] prepared CS/GE/PVA hydrogels for usage in wound-dressing applications. CS/GE/PVA hydrogels showed good pH-sensitivity, swelling ability and water evaporation rate. The dydrogels could adhere to the surface of the wound and absorb blood quickly to block broken blood vessels, while stimulating the platelets to release coagulation factors, which could promote and accelerate the blood coagulation.

### 3.4. CS-Based Composite Hemostatic Particles

In order to improve the hemostatic effects of CS materials, many researchers have fabricated CS hemostats by blending CS with other materials [32,95] and by modifying the physical or chemical structure of CS [24,96]. As with the main compositions, the formulations of hemostasis materials are also essential. As a kind of powdery hemostat, hemostatic particles can be applied to any wounds of shape and depth, which usually cannot be reached by the traditional hemostatic materials such as sponges and films.

**Figure 8:**
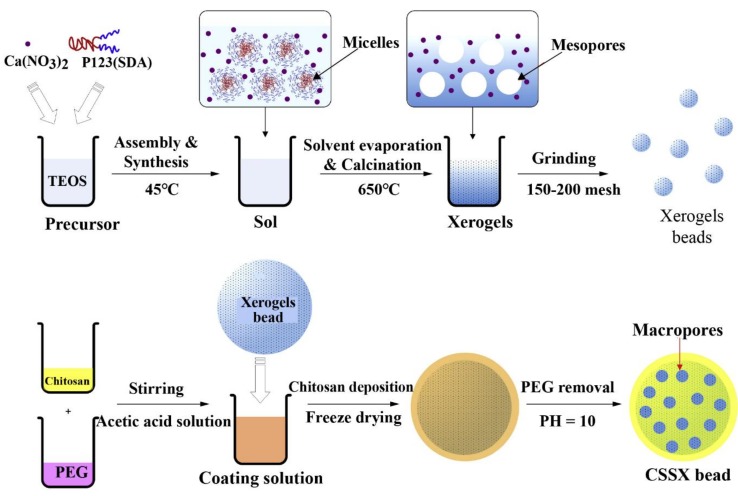
A schematic diagram showing the preparation process of chitosan-silica xerogel (CSSX) beads with macro-mesoporous structure by coating mesoporous silica xerogel cores with macroporous chitosan layers using modified sol-gel process and phase inversion imprinting technique.

QuikClot^®^ zeolite powder (Z-Medica Corporation, Connecticut, USA) has been approved by the FDA and is currently available for use in stopping bleeding in many cases. However, this zeolite-based hemostat is facing the challenging facts of thermal injuries resulting from the exothermic reaction and poor biodegradability [97,98]. The mesoporous silica xerogels (MSX) with large surface area and high porosity developed by Li et al. [99] had great capacity for water absorption with little heat effect when in contact with blood and could be used to staunch bleeding. On this basis, Dai et al. [44] fabricated a series of chitosan-silica xerogel beads (CSSX) with good biocompatibility by combining a liquid-absorbing mesoporous silica xerogel core and a macroporous chitosan coating layer via the modified sol-gel process and polyethylene glycol (PEG) molecular imprinting technique (Figure 8). In vivo and in vitro blood clotting evaluation showed that the CSSX beads could significantly accelerate the contact activation pathway of the coagulation cascade and achieve desirable hemostatic effects. In addition, the CSSX beads could promote the proliferation of mouse myoblast with high cell viability and no cell damage, indicating no cytotoxicity. Thus, the CSSX beads could be used as a safe hemostatic system for further development of functional hemostatic materials.

Microspheres have some advantages for applications in biomedical materials over other particle geometries such as tailored porosity, large surface area, low mass density and excellent cell attachment [100]. Microspheres exhibit good mutual stacking effects, which are particularly important in blood control.

**Figure 9:**
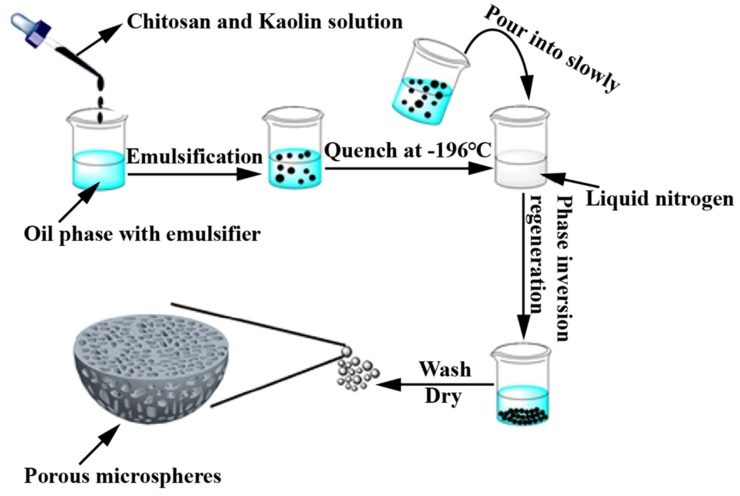
Diagram for the preparation of porous chitosan-kaolin micro-spheres.

Kaolin clay is composed of mineral kaolinite and silicate. The surface charge properties of kaolin have an important effect on its hemostatic capacity. When exposed to plasma, kaolin can activate the coagulation factor XII of the endogenous blood coagulation cascade. To improve the hemostatic effect of CS, Sun et al. [45] fabricated the porous chitosan/kaolin composite microspheres (CSMS-Ks) through a combination of inverse emulsion and thermally induced phase separation (TIPS) techniques (Figure 9). Owing to the synergetic combination of physical (electrostatic adsorption by chitosan) and physiological (activation of the coagulation cascade by kaolin) mechanisms, CSMS-Ks had potent hemostatic potential and were superior to a commercial chitosan hemostat Celox. Histological analysis revealed that CSMS-Ks were safe hemostatic agents and had no obvious adverse effects on the surrounding tissues of the wound. Therefore, CSMS-Ks were promising quick hemostatic agents for traumatic hemorrhaging control.

**Figure 10:**
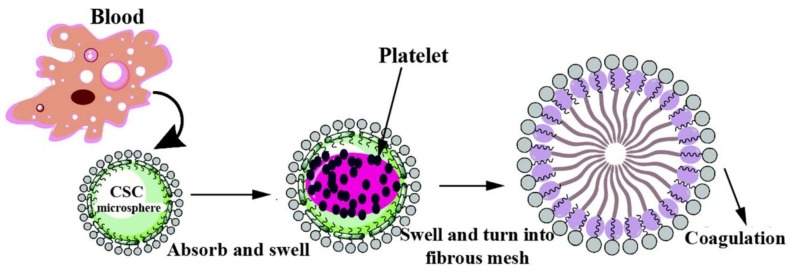
Simplified scheme of the blood clotting process of a composite hemostatic microsphere (CSCM).

Collagen was found to have advantages such as facilitating aggregation of platelets, trigging the intrinsic blood coagulation pathway to facilitate blood clotting, speeding up wound healing, increasing the immunity of the body and reducing wound infection [101,102,103]. Nevertheless, the efficacy of hemostatic materials with one component alone was limited. Compared with CS, carboxymethyl chitosan had better water solubility and lower toxicity. Shi et al. [46] developed novel composite hemostatic microspheres (CSCM) using carboxymethyl chitosan, sodium alginate and collagen as the starting materials. The surface morphology characterization by scanning electron microscopy showed that there were many small promontories on the surface of CSCM, which increased the surface area of blood contact and promoted platelet adhesion. The hemostatic function tests in vitro revealed that CSCM could facilitate adherence, aggregation and activation of platelets. When contacting with blood, activated plasma proteins on the surface of CSCM initiated the cascade of endogenous coagulation, eventually led to thrombin formation. Thrombin transformed fibrinogen to fibrin monomers, which in turn polymerized to form a fibrous net and caused CMCS to swell, thereby achieving blood coagulation (Figure 10). Owing to its active hemostatic function, good biodegradability and non-cytotoxicity, CSCM could be developed to be hemostatic agents for related biomedical applications.

**Figure 11:**
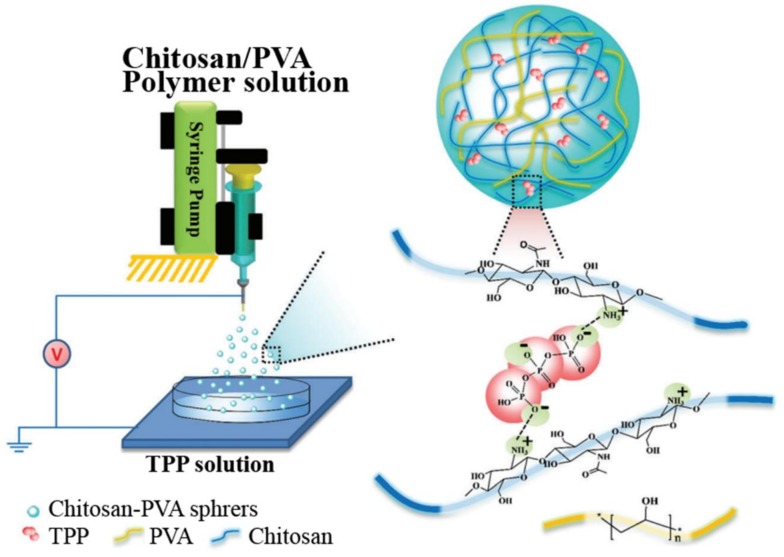
Schematic of chitosan-poly (vinyl alcohol) (PVA) monodisperse spheres fabricated by electrospraying technique and ionotropic gelation.

Poly (vinyl alcohol) (PVA), a hydrophilic material containing a lot of -OH groups, has attracted much attention in biomedical applications, especially in wound healing and tissue engineering due to its unique properties such as good water solubility, outstanding biocompatibility, non-toxicity, excellent hygroscopicity, moisture retention and minimal cell adhesion [104,105]. In view of the dual challenges of efficiency and safety for CS-based hemostatic agents, Chen et al. [47] incorporated the PVA component into CS and fabricated CS-PVA monodisperse millimeter-scale spheres by electrospraying technique and ionotropic gelation (Figure 11). The morphology and swelling property of CS-PVA spheres were characterized and the results showed that the size of spheres increased with the increase of CS content. The swelling properties of the CS-PVA spheres were mainly influenced by the content of PVA. The hemostatic properties of CS-PVA spheres in vitro and in vivo were evaluated by coagulation time assay and rat liver hemorrhage model. CS50-PVA50 spheres showed the best effect and significantly reduced the hemostatic time and blood loss. More importantly, compared with chitosan powders, the spheres also greatly decreased thromboembolic formation in controlling femoral artery bleeding.

### 3.5. CS-Based Fibrous Hemostatic Materials

Nanofibers are similar to the morphology of the natural extracellular matrix in the skin, and have the advantages of high porosity, variable pore size distribution, and high surface area ratio. Therefore, the biomaterials of the nanofiber matrix as skin substitutes have shown great potential. Currently, there are many methods for preparing nanofibers with controllable structure, such as self-assembly, phase separation, electrospinning, and so on. Among these, the electrospinning is a simple, fast, efficient and inexpensive technique which has been widely applied in the preparation of polymer nanofibers by applying a high voltage to an electrically charged liquid [106,107]. Gu et al. [48] fabricated CS-GE nanofiber mats with the electrospinning method. To obtain the porous polymer nanofiber mats, an ultrasonication treatment was used to increase the pore size and thickness of the nanofiber mats. Blood-clotting studies in vitro showed that the hydrophilic gelatin and sonication treatment produced synergetic effects on hemostatic functions including rapid blood absorption and effective blood clotting. Additionally, the sonicated CS-GE nanofiber mats with high porosity supported active cell proliferation, migration and infiltration. Those results demonstrated that the sonicated CS-GE nanofiber mats were promising candidates for hemostatic dressings as well as tissue-engineering scaffolds.

Hemostatic agents need to absorb blood quickly at the early stages of a blood coagulation cascade to achieve rapid and effective control of excessive hemorrhages. For severe bleeding, the form of fiber and non-woven sheets (also referred to as mats) should be the first choice for hemostatic agents because it is easily applied with pressure and removed from the bleeding site. Meanwhile, fiber and non-woven dressings have good permeability and a highly porous microstructure [108,109].

Native batroxobin (nBat), a thrombin-like enzyme with anticoagulation effects, is extracted from *Bothrops atrox* and *Bothrops moojeni* venom and its cDNA is also cloned. Recombinant batroxobin (rBat) interacts with proteins in the coagulation cascade, specifically acting on fibrinolytic pathway, and has a potent procoagulant activity [110]. Chitosan and rBat, which act through different hemostatic mechanisms, were combined and rBat-coated chitosan (rBCC) non-woven dressing was fabricated by Seon et al. [49]. Hemostatic assay in vitro and in vivo showed that the rBCC non-woven dressing facilitated erythrocyte aggregation, fibrin clot formation and blood coagulation in the hemostatic coagulation cascade, resulting in a stable barrier for excessive bleeding, and a synergetic effect was confirmed between the non-woven chitosan and the procoagulant rBat activity.

**Figure 12:**
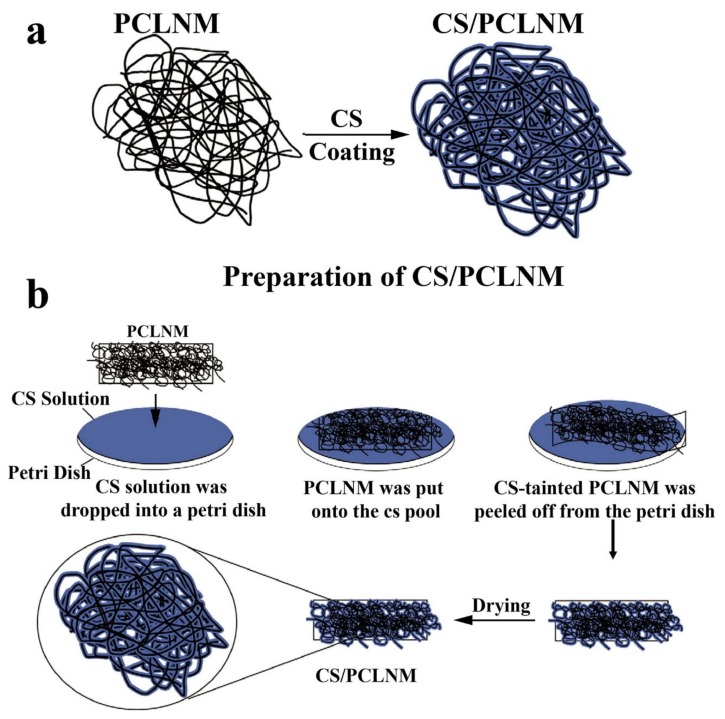
Schematic illustration of the preparation for CS/PCLNM non-woven mat: (**a**) microstructure of the nonwoven mat, and (**b**) a modified drop-casting method for transforming the PCLNM into CS/PCLNM.

For medical materials, the most important feature is to be harmless to the human body. Biodegradable and biocompatible biopolymers are non-toxic to the human body and environmentally friendly. Polycaprolactone (PCL), a semicrystalline polyester, is an ideal biomaterial for medical applications due to its unique properties such as good histocompatibility and biodegradability, lack of antigenicity in humans, a low melting point (55–60 °C), high processability, blend compatibility and low cost [111]. In order to provide an outermost barrier to prevent infection, reduce blood loss, and stop the bleeding, Bai et al. developed a chitosan/polycaprolactone (PCL) non-woven mat (CS/PCLNM) by combining an electrospinning technique and a modified drop-casting method [50]. CS/PCLNM was composed of many microfibers with a CS overlayer on top of the PCL fiber, rather than the CS-PCL blended fiber (Figure 12). Measured by X-ray photoelectron spectroscopy (XPS) and a non-contacting electrostatic voltmeter, CS/PCLNM exhibited a positive potential and the charge density of CS/PCLNM was in proportion to the thickness of the CS overlayer. Functional assays showed that CS/PCLNM possessed multiple pharmacological effects, such as platelet aggregation, anti-bacterial, anti-adhesive and anti-inflammatory activities. Moreover, the performance of CS/PCLNM could be further enhanced by incorporating active compounds such as calcium chloride or tea tree oil. Novel nanofibres were also fabricated via the spraying method by Park et al. [51] using three components including PCL, calcium carbonate (CaCO_3_) and CS. CS-coated PCL/CaCO_3_ nanofibres exhibited high-performance blood coagulation and it was proved that CS played a key role in the change of surface wettability from hydrophobicity to hydrophilicity, which contributed to improving blood coagulability. Thus, CS-coated PCL/CaCO_3_ nanofibres offered promise in medical applications.

Oxidized regenerated cellulose (ORC), in which partial primary hydroxyl groups of cellulose are oxidized to carboxyl groups, is generally used as an absorbable hemostatic agent in surgical operations for low-pressure and diffuse bleeding control [112]. However, due to the relatively poor hemostatic effect and excessive acidic surface, the applications of ORC in the field of hemostasis have been limited. To improve the hemostatic activity of ORC and reduce its acidity, He et al. [52] prepared a water-soluble CS-coated ORC composite (CS/ORC) gauze. The hemostatic assays on rabbit liver and ear-artery injuries showed that CS/ORC gauze had a more significant hemostatic effect compared to ORC. To obtain hemostatic materials which were suitably used for preventing postoperative adhesion in surgery, Cheng et al. [53] developed a *N*,*O*-CS/ORC composite gauze by modifying ORC gauze with *N*,*O*-carboxymethyl chitosan (*N*,*O*-CS) in a nitrogen dioxide/carbon tetrachloride oxidation system. The antibacterial performance of *N*,*O*-CS/ORC composite gauze increased as the content of *N*,*O*-CS increased. Hemostatic evaluation in the rabbit liver hemostasis model indicated that *N*,*O*-CS/ORC composite gauze had excellent hemostatic effects. These results showed that *N*,*O*-CS/ORC composite gauze had great potential to be applied for hemostasis and preventing post-operative adhesion.

### 3.6. Other CS-Based Composite Hemostatic Materials

Porous silica with a porous structure can fast absorb plasma without obvious associated exothermic reactions and is a novel, promising hemostatic material. Currently, many inorganic mesoporous materials, such as spherical bioactive glass [69], mesocellular silicate foams [113] and mesoporous silica spheres [68], were used to study for bleeding control and exhibited excellent hemostatic effects. However, due to lack of safety, unavailability and high cost, the applications of these synthetic mesoporous silicas in the field of biomedicine were severely limited. Diatom silica, produced by a biosynthesis process with single cellular microalgae, had excellent advantages and presented strong potential to replace synthetic silica-based materials [114,115,116]. To improve the biocompatibility and hemostatic activity of diatom silica, Feng et al. [54] prepared a series of CS-coated diatom (CS-diatom) with chitosan and natural diatom silica which was obtained from diatomite and diatom culture. The CS-diatom prepared with 1% CS had favorable biocompatibility and great fluid absorbability, which could stop bleeding by fast absorbing the plasma effusion and inducing the absorption and aggregation of red blood cells. Compared with gauze and the commercial QuikClot zeolite (Z-Medica Corporation), CS-diatom had the shortest blood-clotting time and the lowest amount of blood loss in a rat-tail amputation model. These results showed that the CS-diatom as a non-synthetic mesoporous silica composite material was safe and effective for hemorrhage control.

In view of the dual challenges of bleeding and contamination in wounds, a chitosan-based wound dressing by incorporating a procoagulant (polyphosphate) and an antimicrobial (silver) was fabricated by Ong et al. and its hemostatic efficacies were evaluated in vitro [55]. The results demonstrated that the chitosan-polyphosphate (ChiPP) dressing accelerated blood coagulation, platelet adhesion and thrombin generation. It absorbed more blood to stop bleeding more quickly than chitosan. The silver-loaded ChiPP (ChiPP-Ag) dressing showed stronger antibacterial activity than ChiPP did in vitro, achieving a complete kill of *Pseudomonas aeruginosa* (*P. aeruginosa*) and a > 99.99% kill of *Staphylococcus aureus*. In a full-thickness wound model contaminated with high levels of *P. aeruginosa*, the ChiPP-Ag dressing significantly decreased the mortality rate from 90% to 14.3% compared to standard gauze treatment. For more extensive and effective applications, multiple forms of ChiPP-Ag (e.g., microspheres, hydrogels) can be designed to accommodate varied wound types and improve silver delivery to the wound.

Superabsorbent polymers, a class of polymers with network structures, can absorb a large amount of water that is hundreds or even thousands of times heavier than their mass [117]. Due to their high swelling capacity and potential hemostatic ability, superabsorbent polymers have attracted considerable attention. The superabsorbent polymers fabricated from CS and its derivatives were potential hemostatic materials with good water absorption and excellent hemostatic properties [118,119]. By using a solvent precipitation method, Chen et al. [56] prepared a porous carboxymethyl chitosan-grafted poly (acrylic acid) (CMCTS-g-PAA) superabsorbent polymer. The investigated results showed that CMCTS-g-PAA superabsorbent polymer possessed high swelling capacity and was non-cytotoxic. In the hemorrhage model of an ear artery, arteria cruralis and spleen of rabbits, CMCTS-g-PAA superabsorbent polymer exhibited an excellent hemostatic performance, which was the result of the synergistic effects among the positive charge of CS, the strong swelling capacity, and rough surface of the porous polymer structure.

## 4. Future Perspectives 

With the development of medical services, the requirements of the performance of hemostatic materials are increasing. It is of great significance to fabricate fast, efficient, safe and ready-to-carry novel hemostatic materials. Thus, it is necessary to study systematically the hemostatic mechanisms and synergistic effects of CS and CS-based hemostatic materials. Various hemostatic materials with different hemostasis mechanisms may be incorporated together to give full play to the advantages of different materials, increase hemostasis pathways, accelerate the hemostasis speed, and eventually achieve fast hemostasis. Meanwhile, the hemostatic effects of hemostats vary with their forms. The forms of CS-based composite materials can be optimized to obtain fast hemostasis. In addition, CS can be blended with other functional components such as pain relieving, anti-inflammatory and wound healing materials to obtain CS-based multi-functional composite hemostatic materials.

## Figures and Tables

**Table 1:** The summary of chitosan-based composite materials for hemostasis.

Materials	Major components	Characteristics	Ref.
Films	CS, gelatin (GE), ibuprofen	High swelling degree, antibacterial activity, no obvious cytotoxicity and haemorrhage reducing.	[28]
	CS, alginate (AG), *Yunnan Baiyao*	Good hemostatic performance and producing synergetic effects.	[29]
	CS, mesoporous bioactive glass (MBG)	High porosity, good degradability, biocompatibility, water adsorption and non-cytotoxicity.	[30]
	CS, Ga-MBG	High hemostatic efficacy, facilitating cell proliferation and excellent antibacterial activity.	[31]
Sponges	CS, GE	Promoting cell proliferation, no significant cytotoxicity, obvious bacteriostatic effects and good biodegradability.	[32]
	CS, squid ink polysaccharide (SIP)	Strong absorptivity, significant procoagulant effects and promoting wound healing.	[33]
	Chitin, SIP	No significant effects on the blood parameters including coagulation, anticoagulation, fibrinolytic and hemorheology.	[34]
	CS, hydroxybutyl chitosan	High porosity, great water absorption, no cytotoxicity excellent antibacterial properties, and making the blood form viscous gels which were conducive to promoting blood coagulation.	[35]
	CS, oxidized nanofibrillar cellulose	Superior biocompatibility and biodegradability, fast absorption of blood and non-cytotoxicity.	[36]
	CS, sodium polyacrylate (SPA), polyethylene glycol	Good water absorbency, superior hemostatic effects, wound sealing and external pressure application.	[37]
	CS, calcium pyrophosphate, Col	Rapid water absorption ability, high specific surface area, activating the intrinsic pathway of coagulation cascade, and complete biodegradation in three weeks.	[38]
	CS, poly-(methyl vinyl ether-co-maleic anhydride)	Good porosity, and strong clotting ability.	[39]
Hydrogels	Thiol functionalized chitosan, maleimide group modified ε-polylysine	Non-toxicity, excellent hemostatic property, and high adhesion strength.	[40]
	Catechol-functionalized chitosan, thiol-terminated Pluronic	Superior mechanical performance and stability, strong adhesiveness, excellent hemostatic properties, injectable and thermosensitive properties	[41]
	Poly (vinyl alcohol) (PVA), human-like collagen, carboxymethyl chitosan (CMCS)	Good swelling ability, hemostatic and bacterial barrier activities, biocompatibility and wound healing.	[42]
	CS, GE, PVA	Good pH-sensitivity, swelling ability, water evaporation rate and adhesion.	[43]
Particles	CS, mesoporous silica xerogels	Promoting the cell proliferation, no cytotoxicity; great capacity for water absorption, and accelerating the contact activation pathway of coagulation cascade.	[44]
	CS, Kaolin clay	High amount of pores, no adverse effects, and the synergetic combination mechanisms.	[45]
	CMCS, AG, Col	Facilitating platelet adherence, aggregation and activation, high water absorption ability, good biodegradability and non-cytotoxicity.	[46]
	CS, PVA	Significant reduction of the hemostatic time and blood loss, narrow size distribution and good biocompatibility.	[47]
Fibers	CS, GE	High porosity and wettability, rapid blood absorption and effective blood clotting.	[48]
	CS, recombinant batroxobin	Facilitating erythrocyte aggregation, fibrin clot formation and blood coagulation.	[49]
	CS, polycaprolactone (PCL)	Possessing multiple pharmacological effects, such as platelet aggregation, anti-bacterial, anti-adhesive and anti-inflammatory activities	[50]
	CS, PCL, CaCO_3_	High-performance blood coagulation.	[51]
	CS, oxidized regenerated cellulose (ORC)	Good antibacterial and degradable properties, forming a gel by absorbing blood and then sealing off the crevasses of the blood vessels to stop bleeding.	[52]
	CMCS, ORC	Excellent hemostatic effects and preventing post-operative adhesion.	[53]
Others	CS, natural diatom silica	Favorable biocompatibility, great fluid absorbability, no cytotoxicity and desirable hemostasis effects.	[54]
	CS, polyphosphate	Strong antibacterial activity, accelerating blood clotting, platelet adhesion and thrombin generation, and decreasing the mortality rate in a wound model.	[55]
	CMCS, poly (acrylic acid)	Excellent hemostatic performance, good swelling capacity and non-cytotoxic.	[56]

## Abbreviations

AG	Alginate
ChiPP	Chitosan-polyphosphate
CMCS	Carboxymethyl chitosan
Col	Collagen
CS	Chitosan
CS-C	Catechol-functionalized chitosan
CSS	Thiol functionalized chitosan
EPLM	Maleimide group modified ε-polylysine
GE	Gelatin
HBC	Hydroxybutyl chitosan
HLC	Human-like collagen
HM-CS	Hydrophobically modified chitosan
MBG	Mesoporous bioactive glass
MSX	Mesoporous silica xerogels
ONFC	Oxidized nanofibrillar cellulose
ORC	Oxidized regenerated cellulose
PAA	Poly (acrylic acid)
PCL	Polycaprolactone
PEG	Polyethylene glycol
Plu-SH	Thiol-terminated Pluronic
PVA	Polyvinyl alcohol
PVM/MA	Poly-(methyl vinyl ether-co-maleic anhydride)
rBat	Recombinant batroxobin
RBC	Red blood cell
SIP	Squid ink polysaccharide
SPA	Sodium polyacrylate
